# Deletion of Clusterin Protects Cochlear Hair Cells against Hair Cell Aging and Ototoxicity

**DOI:** 10.1155/2021/9979157

**Published:** 2021-05-29

**Authors:** Xiaochang Zhao, Heidi J. Henderson, Tianying Wang, Bo Liu, Yi Li

**Affiliations:** ^1^Department of Otorhinolaryngology Head and Neck Surgery, Beijing Tongren Hospital, Capital Medical University, Beijing, China; ^2^Department of Biomedical Sciences, Creighton University School of Medicine, Omaha, Nebraska 68178, USA; ^3^Beijing Institute of Otolaryngology, Key Laboratory of Otolaryngology Head and Neck Surgery (Capital Medical University), Ministry of Education, Beijing, China

## Abstract

Hearing loss is a debilitating disease that affects 10% of adults worldwide. Most sensorineural hearing loss is caused by the loss of mechanosensitive hair cells in the cochlea, often due to aging, noise, and ototoxic drugs. The identification of genes that can be targeted to slow aging and reduce the vulnerability of hair cells to insults is critical for the prevention of sensorineural hearing loss. Our previous cell-specific transcriptome analysis of adult cochlear hair cells and supporting cells showed that *Clu*, encoding a secreted chaperone that is involved in several basic biological events, such as cell death, tumor progression, and neurodegenerative disorders, is expressed in hair cells and supporting cells. We generated *Clu*-null mice (C57BL/6) to investigate its role in the organ of Corti, the sensory epithelium responsible for hearing in the mammalian cochlea. We showed that the deletion of *Clu* did not affect the development of hair cells and supporting cells; hair cells and supporting cells appeared normal at 1 month of age. Auditory function tests showed that *Clu*-null mice had hearing thresholds comparable to those of wild-type littermates before 3 months of age. Interestingly, *Clu*-null mice displayed less hair cell and hearing loss compared to their wildtype littermates after 3 months. Furthermore, the deletion of *Clu* is protected against aminoglycoside-induced hair cell loss in both *in vivo* and *in vitro* models. Our findings suggested that the inhibition of *Clu* expression could represent a potential therapeutic strategy for the alleviation of age-related and ototoxic drug-induced hearing loss.

## 1. Introduction

Hearing loss is one of the most common sensory impairments in humans, affecting approximately 1.3 billion people worldwide [1]. Sensorineural hearing loss, which accounts for a large proportion of hearing loss, is permanent and often caused by acoustic trauma, ototoxic drugs, aging, environmental factors, and genetic defects. Hearing loss affects speech understanding [2], which can lead to isolation, depression, and dementia [3]. However, to our knowledge, no drugs have been identified that can treat or prevent sensorineural hearing loss. The hair cells in the cochlea of the mammalian inner ear are mechanosensitive receptor cells that transduce mechanical stimuli into electrical signals. Two types of hair cells exist in the cochlea. The inner hair cells (IHCs) are the actual sensory receptors that relay electrical signals to the central auditory system through the spiral ganglion neurons, whereas outer hair cells (OHCs) amplify the mechanical signals in the cochlea [4]. Cochlear supporting cells maintain homeostasis of the ionic and chemical environment of the cochlea as well as contribute to the stiffness of the cochlear partition. Supporting cell defects can also lead to hair cell degeneration and hearing loss [5]. All hair cells, including those in the inner ear of nonmammals, are vulnerable to aging, noise, and ototoxicity drugs. The molecular mechanisms that underlie hair cell aging and vulnerability to mechanical and chemical insults remain unclear [6].

Clusterin (CLU) is a secreted chaperone protein involved in several basic biological events, such as cell death, tumor progression, and neurodegenerative disorders. CLU is expressed in many tissues in humans and animals, such as the testis, epididymis, kidneys, heart, lungs, uterus, ovary, breast, and prostate [7]. Moreover, CLU has been found in the bodily fluids of almost all vertebrates, from zebrafish to humans [8, 9]. CLU plays important roles in protein homeostasis/proteostasis, the inhibition of cell death pathways, and the modulation of prosurvival signaling and transcriptional networks [10]. Previous studies have shown that CLU is associated with neurodegenerative diseases, immune diseases, aging, and cancer [10].

Our previous study used cell type-specific RNA sequencing (RNA-seq) analyses which show that *Clu* is expressed in hair cells and supporting cells [11, 12]. In the present study, we generated a *Clu-*null mouse model to examine the role of CLU in the inner ear. We compared changes in inner ear morphology and function between *Clu*-null and wild-type (WT) mice. We showed that deletion of *Clu* did not affect the development of cochlear hair cells and supporting cells; however, *Clu* deficiency delayed the onset and progression of age-related hearing loss (ARHL) and reduced aminoglycoside-induced hair cell and hearing loss.

## 2. Materials and Methods

### 2.1. Ethics Statement

**Figure 1:**
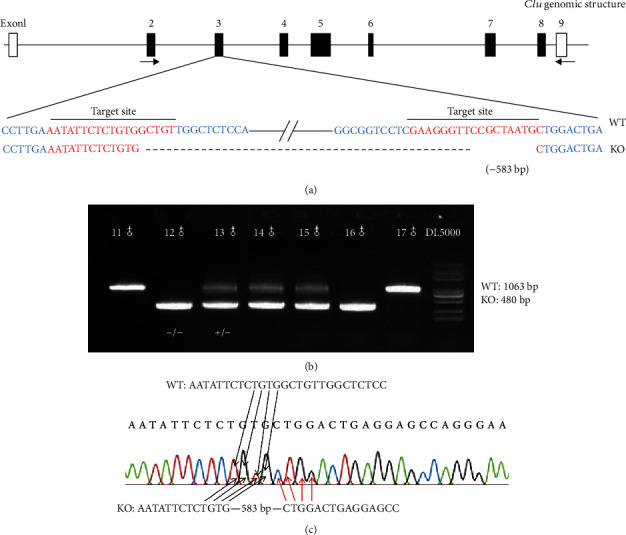
Construction of *Clu* knockout mice. (a) Schematic drawing showing the strategy used for *Clu* gene disruption. The target sites of clustered regularly interspaced short palindromic repeat (CRISPR)-Cas9 small guide RNAs (sgRNAs) in the *Clu* gene are indicated in red, and the deleted region of the *Clu* gene in knockout mice is indicated by dashes. The positions of RT-PCR primers are indicated by arrows. (b) PCR products were separated on a 1.5% agarose gel, and the expected band sizes for WT and knockout (KO) alleles were 1,063 and 480 bps, respectively. (c) Sequencing results for homozygotes. The black arrows represent wild-type sequences, and the red arrows represent sequences following the deleted fragment.

All experimental procedures were approved by the Animal Ethics Review Committee of Capital Medical University. The usage of animals followed the Guide for Use of Laboratory Animals of the Capital Medical University in Beijing, China. *Clusterin* Knockout Mice and Genotyping *Clusterin* knockout mice were generated on C57BL/6 background by CRISPR/Cas9 technology. The *Clu* was deleted by replacing exon 3 of the *Clu* gene, which results in a 583 bp deletion. Genomic DNA was extracted from the tails of the newborn pups (*N* = 4 each group). The genomic DNA fragment around the guide RNA target site was amplified by *Clu*-KO-specific PCR primers as the following primer sets: forward:5′-CAACCGCATCAGGTAGA-3′; reverse:5′-ACCCAC AGCAAGGGTTAG-3′. F0 mice were bred to generate F1 mice. The PCR cycling parameters were as follows: 94°C for 3 min; 35 cycles of 94°C for 30 s, 56°C for 60 s, 72°C for 60 s; 72°Cfor 10 min. PCR products were separated on 1.5% agarose gel, and the expected band sizes for WT (wild-type) and knockout alleles were 1063 and 480 bps, respectively (Figure 1(b)). The DNA from the F1 offspring generation was sequenced to verify the mutation (Figure 1(c)). F2 generation was obtained by intercrossing heterozygous F1 offspring.

### 2.2. Auditory Brainstem Response (ABR) Measurements

ABR thresholds were measured with tone burst stimuli at frequencies of 8, 16, and 32 kHz in a sound-isolated chamber using a Tucker-Davis Technologies System workstation (RZ6) with SigGen32 software (Tucker-Davis Technologies Inc., Alachua, FL, USA). Mice were anesthetized with a ketamine/xylazine (18 : 2 mg/ml) solution (100/10 mg/kg body weight) and then placed on a soft pad. Subcutaneous electrodes were placed between the ears at the forehead for noninverting, underneath the left external ear for inverting, and back near the tail for ground lead. Auditory brainstem response (ABR) intensity series were collected with a descending series of stimulus levels in 5 dB steps beginning at 90 dB SPL. At least 6 mice per group/age were used for ABR hearing assessment. Following the ABR hearing measurements, tissues from the same mice were used to conduct histopathological and biochemical analyses.

### 2.3. Distortion Product Otoacoustic Emission (DPOAE) Measurements

DPOAE responses were recorded from anesthetized mice using TDT RZ6. The cubic (2f1-f2) DPOAE thresholds were obtained at a range of frequencies (4-32 kHz), evoked using two equal intensity primary stimuli about the test frequency, and applied to the ear canal using two speakers coupled via a microphone probe. Stimuli were decreased in intensity from 80 to 20 dB in 5 dB increments to establish thresholds. The threshold of DPOAE was defined as 6 dB above the noise floor. We used at least 6 mice per group for DPOAE assessment.

### 2.4. Cochlear Tissue Processing

After ABR testing, the mice were sacrificed by cervical dislocation and decapitation, and the cochleae were quickly separated from the temporal bone on the culture dish with 4% paraformaldehyde solution. Under a dissecting microscope, a hole was poked at the apex of the cochlea, and the round and oval windows were opened with a needle. The cochlea was perfused with 4% paraformaldehyde solution instead of lymph fluid via the apex and preserved with 4% paraformaldehyde solution at 4°C overnight. Then, the specimen was decalcified in 10% ethylenediaminetetraacetic acid (EDTA) solution for 1.5 hours. The cochlea shell was removed from the apex to base in PBS solution. The basilar membrane was harvested without the vestibular membrane and tectorial membrane. Six cochleae from 6 mice were used for histopathological assessment for each group/age.

### 2.5. Immunostaining and Laser Confocal Microscopy

The samples were washed three times in PBS and preincubated for 1 hour at room temperature in a blocking solution of 10% normal goat serum in 0.01 M PBS with 0.25% Triton X-100. Next, the samples were incubated with a combination of antibodies about rabbit antimyosin VIIa (1 : 300, Proteus Biosciences, 25-6790), left at 4°C overnight. After incubation, the samples were washed in PBS three times and incubated with the appropriate secondary antibodies coupled to Alexa Fluor in green, red channels at room temperature for 1 hour. After incubation, the samples were washed in PBS three times, and approximately 40 *μ*L of DAPI (4, 6-diamidino-2-phenylindole; Santa Cruz) was applied for the nucleus staining.

Cochlear tissues were fixed with 4% paraformaldehyde in PBS overnight at 4°C. The specimens were decalcified over 12 h in 10% EDTA. The preparations then were dehydrated with graded alcohol series, cleared with xylene, and then embedded in paraffin. Paraffin-embedded specimens were cut into 10-*μ*m-thick sections and then stained with H&E. Confocal images were acquired using Leica confocal microscope (TCS SP8 II; Leica Microsystems, Wetzlar, Germany). 20x magnification was used for imaging and cell counting.

We used Myosin VIIa to label hair cells and DAPI to mark the nucleus of supporting cells underneath the hair cells for supporting cell count. To make cell count results comparable between mice of different ages and genotypes, we compared the actual number of supporting cells to the number of hair cells in the same cochlear location to obtain their relative percentages.

### 2.6. Drug Administration *In Vivo*

6-week-old mice were divided into three groups: control group, WT group, and *Clu^−/−^* group (*N* = 6 for each group). In the control group, mice were given a subcutaneous injection of saline followed by an intraperitoneal injection of saline 30 minutes later. In the other two groups, mice were injected subcutaneously with kanamycin (0.1 mg/g of body weight; Sigma-Aldrich, St Louis, Missouri), dissolved in phosphate-buffered saline (PBS), followed by an intraperitoneal injection of furosemide (0.3 mg/g of body weight) 30 minutes later. For each group, ABR threshold was measured before injection and 10 days after coadministration of kanamycin and furosemide injection.

### 2.7. Organotypic Culture of the Organ of Corti and Drug Administration

Three-day-old mice were divided into three groups (control group, WT group, and *Clu^−/−^* group (*N* = 6 each group) for culture. The basilar membrane together with the organ of Corti were dissected out and place on the bottom of culture dish as previously described [12] in a humidified CO_2_ incubator at 37°C. After 24 hours, the culture medium in the WT group and *Clu^−/−^* group was replace with fresh DMEM/F12 mediums containing 150 *μ*M gentamicin, while the control group was replaced with DMEM/F12 medium. The culture continued for another 36 hours.

### 2.8. Small Molecule Fluorescent *In Situ* Hybridization

Cochleae from 21-day-old (C57BL/6) mice were dissected out and placed in 4% PFA in PBS at room temperature for 24 hours, followed by decalcified in 120 mM EDTA in 4%PFA at 4°Cfor 3 days until the bony tissue was soft and flexible. The tissues were washed in PBS, dehydrated using a standard ethanol series, followed by xylene. After the cochlear wall was removed, the tissues were embedded in paraffin. After paraffin embedding, the blocks were allowed to harden overnight before the tissues were sectioned along the perpendicular plane on a Leica microtome into 5-10 *μ*m sections and mounted on Superfrost plus slides.

To examine the mRNA level expression of *Clu*, the RNAscope-based small molecule fluorescent *in situ* hybridization (smFISH) assay from Advanced Cell Diagnostics (ACD) was used. The paraffin-embedded sections mounted on the slides were baked for one hour at 60°C followed by deparaffinization step (xylene 5 min. 2x, 100% ethanol for 1 min. 2x, and dried at room temperature for 5 min.). A minimum of two sections from different cochlear regions were selected for smFISH. The tissue sections were covered with 5 to 8 drops of RNAscope hydrogen peroxide and incubated at room temperature for 10 min, followed by two washes in fresh distilled water. Slides were dried at room temperature, and a barrier was drawn around each tissue sample using an Immedge hydrophobic barrier pen. The protocol was conducted following the RNAscope 2.5 HD Detection Reagent, RED user manual, and published protocols. The proprietary gene-specific probe for *Clu* targets region n. 436 to 1335 (20 probe pairs). After completing the hybridization, amplification, and color detection steps, the slides were incubated with DAPI for 10 minutes. Finally, the slides were washed in 1X PBS and allowed to dry at room temperature. A drop of SlowFade (Invitrogen) was placed over the tissue and a coverslip was added to preserve the sample. Slides were imaged on a Zeiss LSM 710 confocal microscope. Images were analyzed using ImageJ.

### 2.9. RNA Extraction and Quantitative Real-Time Polymerase Chain Reaction (qRT-PCR)

Total RNA was extracted from the inner ear of C57BL/6 wild-type mice using the RNeasy Mini kit (Tiangen, China). The RNase-free DNase set (Tiangen) was used to remove contaminated DNA. RNA concentration and purity were estimated via spectrophotometry by measuring the absorbance at 260 nm and 260/280 nm ratio, respectively. cDNA was synthesized from RNA using the High Capacity cDNA Reverse Transcription Kit (KAPA BIOSYSTEMS, USA) and oligo-dT primers. Subsequently, qRT-PCR was performed to analyze *Clu* expression at postnatal days 3 (P3), 12 (P12), 30 (1 m), and 180 (6 m). Four mice per group/age were used. qRT-PCR primers were designed based on published reference sequences in the National Center for Biotechnology Information database (NM_013492.3). qRT-PCR was performed using the KAPA SYBR®FAST qPCR Kit Master Mix (2X) Universal (KAPA BIOSYSTEMS) on a StepOnePlus Real-Time PCR System. Each sample was run in triplicate along with the housekeeping gene, GAPDH. Relative quantities of the transcripts were determined using 2^−△△CT^ method using GAPDH as a reference.

### 2.10. Statistical Analyses

Data have been expressed as means ± standard deviation. Statistical analyses were performed with SPSS 13.0 software (IBM, Chicago, IL, USA). A one-way analysis of variance was performed to compare groups. Differences between groups with *p* < 0.05 were considered statistically significant.

## 3. Results

### 3.1. *Clu* Expression in the Organ of Corti

**Figure 2:**
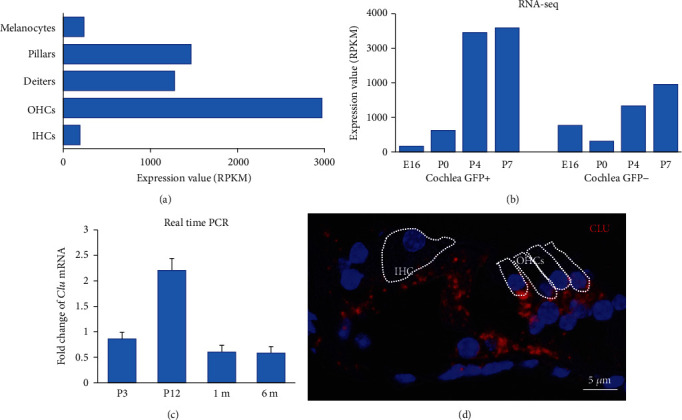
*Clu* expression in the organ of Corti of the cochlea. (a) RNA-seq in the adult CBA/J mouse cochlea showed *Clu* expression in OHCs, IHCs, pillar cells, Deiters' cells, and melanocytes, with strong expression in OHCs. (b) RNA-Seq examining gene expression during mouse inner ear development showed *Clu* expression in hair cells (GFP^+^) and supporting cells (GFP^−^) in the mouse cochlea, from E16 to P7. (c) Temporal expression patterns of *Clu* mRNA in the mouse inner ear. *Clu* mRNA transcripts were strongly expressed at P12, with slight but significantly decreased expression with aging. *N* = 4. (d) *In situ* hybridization performed in the WT cochlea at P21 showed *Clu* expression in OHCs and stronger expression in the pillar and Deiters' cells.

We first examined the expression of *Clu* in the organ of Corti based on published transcriptomes from OHCs, IHCs, pillar cells, Deiters' cells, and stria melanocytes [11, 12]. As shown in Figure 2(a), *Clu* is expressed in hair cells and supporting cells.  Figure 2(b) presents the expression of *Clu* in developing hair cells and supporting cells based on the dataset published by Scheffer et al. [13]. *Clu* was expressed in developing hair cells and supporting cells. To confirm the temporal patterns of *Clu* expression in the mouse inner ear, we performed quantitative real-time polymerase chain reaction (qRT-PCR) at P3, P12, P1m, and P6m. *Clu* mRNA transcripts were strongly expressed at P12, which slightly decreased with aging (i.e., at 1 m and 6 m) (Figure 2(c)). We also used smFISH to examine the spatial expression of *Clu* in the cochlea at P21. As shown in Figure 2(d), *Clu* was expressed in OHCs, but stronger expression was observed in the pillar and Deiters' cells.

### 3.2. Auditory Function of *Clu*^−/−^ Mice

**Figure 3:**
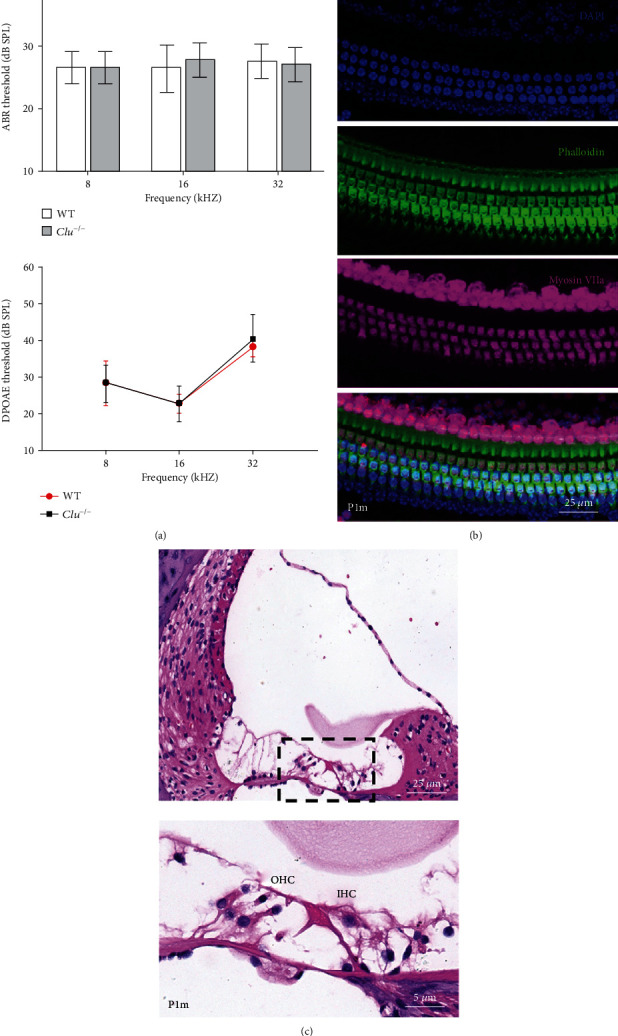
Hearing and basilar membrane morphology of *Clu*^−/−^ mice in one month. (a) ABR hearing thresholds and DPOAE hearing threshold were measured at 32, 16, and 8 kHz in *Clu^−/−^* and WT mice at P1m. *N* = 6. (b) Cochlear basilar membrane morphology of *Clu*^−/−^ mice at P1m. (c) Organ of Corti in the basal cochlear regions of P1m *Clu^−/−^* mice. No hair cells or supporting cell defects were observed.

We measured ABR from *Clu*^−/−^ and their WT littermates at P15 and P30.  Figure 3(a) shows the mean ABR thresholds at 8, 16, and 32 kHz. We also measured DPOAE thresholds from these animals. It is clear from Figure 3(a) that the ABR and DPOAE thresholds of the *Clu^−/−^* mice are not statistically different from those of the WT mice. This indicated that deletion of *Clu* did not affect the development of hair cells and auditory function at 1 month of age.

### 3.3. Hair Cells and Supporting Cell Morphology in *Clu*^−/−^ Mice

To investigate the morphology of the organ of Corti of *Clu*^−/−^ mice, we performed histological analyses on cochlear sections from WT and *Clu*^−/−^ mice at P3, P15, and P1m. Some examples are presented in Figure 3. We counted the total number of hair cells and found the numbers of hair cells and supporting cells in *Clu*^−/−^ mice were not significantly different from the WT mice. Furthermore, we examined morphology of the organ of Corti in the basal turn in P15 and 1 m-old *Clu^−/−^* mice using HE staining. No defects were observed in either the hair cells or supporting cells in the *Clu^−/−^* cochleae. Thus, deletion of *Clu* did not affect the development of hair cells and supporting cells.

### 3.4. *Clu* Deficiency Delays Onset and Progression of Age-Related Hearing Loss

**Figure 4:**
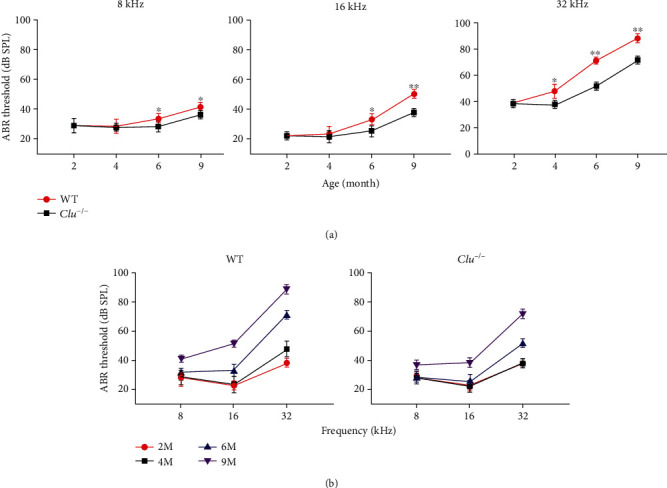
Assessment of age-related auditory dysfunction. (a) ABR hearing thresholds were measured at 32, 16, and 8 kHz in WT and *Clu^−/−^* mice at P2m, P4m, P6m, and P9m. *Clu* deficiency delayed ARHL in C57BL/6 mice. Data are presented as the mean ± SD. ^∗^*p* < 0.05, ^∗∗^*p* < 0.01. *N* = 6. (b) ABR thresholds of WT and *Clu^−/−^* mice at P2m, P4m, P6m, and P9m. *N* = 6.

To investigate whether *Clu* plays a role in the maintenance of auditory function or ARHL, we conducted ABR tests in WT and *Clu*^−/−^ mice at 2, 4, 6, and 9 months of age. The ABR thresholds at 8, 16, and 32 kHz are presented in Figure 4. It is apparent that the ABR thresholds of WT at 16 and 32 kHz were elevated with age. Interestingly, the threshold elevation of *Clu*^−/−^ mice at 16 and 32 kHz was significantly less comparing to that of the WT mice. This suggests that deletion of *Clu* has altered the onset and progression of ARHL.

**Figure 5:**
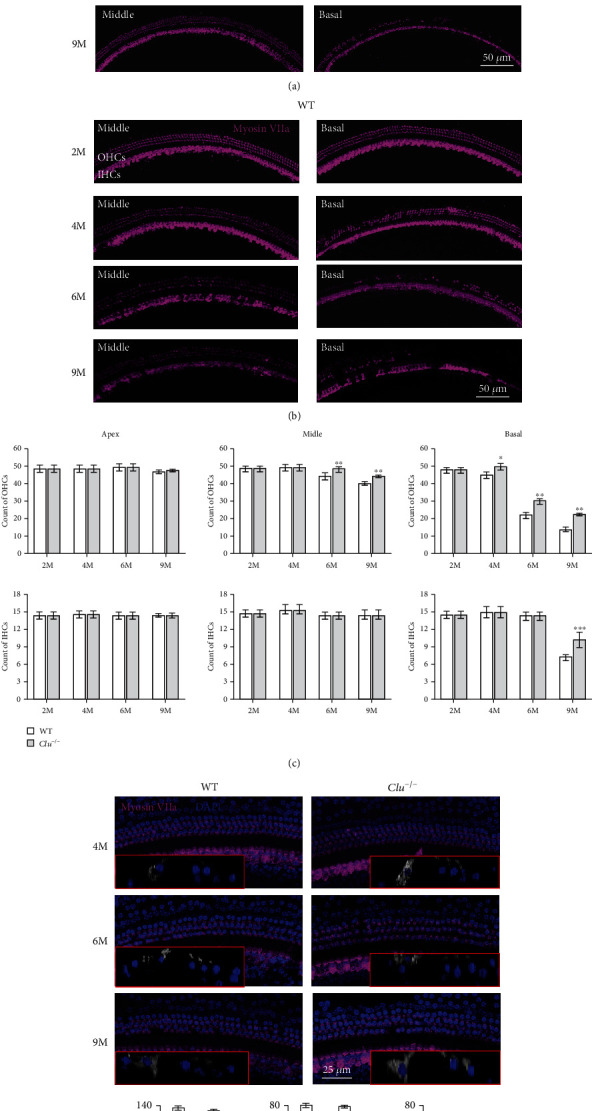
Assessment of age-related morphology of hair cells and supporting cells. (a) Cochlear basilar membrane immunostaining in P2m, P4m, P6m, and P9m *Clu^−/−^* mice. (b) Cochlear basilar membrane immunostaining in P2m, P4m, P6m, and P9m WT mice. The number of remaining hair cells in *Clu^−/−^* mice was significantly higher than that in WT mice at the same age. (c) Quantification of OHCs and IHCs in mice at P2m, P4m, P6m, and P9m across all tested frequencies in WT and *Clu^−/−^* mice. Data are presented as the mean ± SD. ^∗^*p* < 0.05, ^∗∗^*p* < 0.01. *N* = 6. (d) Quantification of supporting cells at P4m, P6m, and P9m in WT and *Clu^−/−^* mice. The red box shows a typical section after three-dimensional reconstruction. The percentages of supporting cells and hair cells showed no significant differences between WT and *Clu^−/−^* mice. *N* = 6.

ARHL in C57BL/6 mice develops as a result of the degeneration of sensory hair cells [2, 14–16]. We performed histological analyses on cochlear sections from WT and *Clu*^−/−^ mice at 2, 4, 6, and 9 months. The cochleae from WT and *Clu^−/−^* mice at 2 m displayed no evidence of IHC or OHC loss. At 4 m, the basal region of the cochleae from WT mice displayed severe loss of OHCs, whereas the basal region of the cochlea from *Clu^−/−^* mice displayed no loss of hair cells. At 6 and 9 months, both WT and *Clu^−/−^* mice showed hair cell loss. However, the number of remaining OHCs in *Clu^−/−^* mice was significantly higher than that of the WT mice, in both the middle (*p* < 0.05) and basal turn (*p* < 0.05) of the cochleae. We also counted the number of Deiters' cells from WT and *Clu*^−/−^ mice at these ages. The number of Deiters' cells was comparable that of OHCs (Figure 5(d)).

### 3.5. *Clu* Deficiency Protects Hair Cell Loss against Ototoxicity Induced by Coadministration of Kanamycin and Furosemide

**Figure 6:**
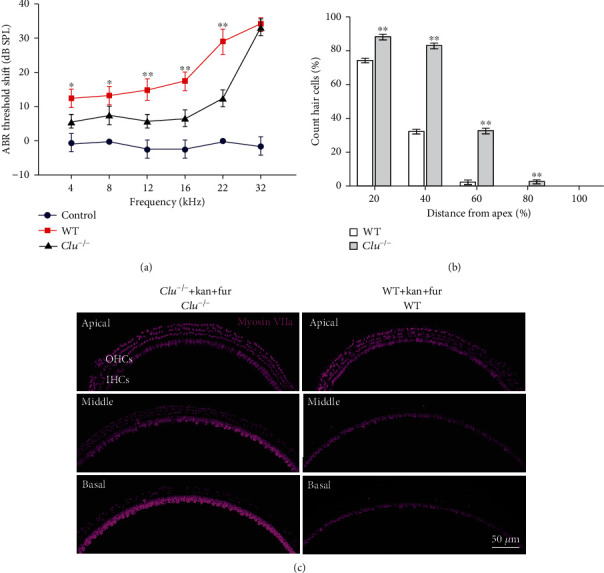
*Clu* deficiency protects hearing ability and inhibits sensory cell death induced by the coadministration of kanamycin and furosemide. (a) Assessment of auditory dysfunction in WT and *Clu^−/−^* mice treated with furosemide plus kanamycin. *N* = 6. (b) Quantification of OHCs and IHCs in WT and *Clu^−/−^* mice after treatment with furosemide plus kanamycin. Data are presented as the mean ± SD. ^∗^*p* < 0.05, ^∗∗^*p* < 0.01. *N* = 6. (c) Representative confocal microscopy images from WT and *Clu^−/−^* mice after treatment with furosemide plus kanamycin.

Both WT and *Clu^−/−^* mice were treated with the combination of kanamycin and furosemide at 7–8 weeks to examine drug-induced hearing loss. Before drug treatment, the ABR threshold was measured to ensure that all mice used had normal auditory function.  Figure 6 shows the ABR threshold shifts of WT and *Clu^−/−^* mice treated with furosemide and kanamycin. The threshold shifts were calculated by subtracting the ABR thresholds of the untreated control WT mice from the ABR thresholds of the treated WT and *Clu^−/−^* mice. The results showed that 10 days after drug treatment, the ABR threshold shifts of WT mice were significantly greater than the *Clu^−/−^* mice, suggesting that *Clu* deficiency protected hearing loss against furosemide and kanamycin ototoxicity.

**Figure 7:**
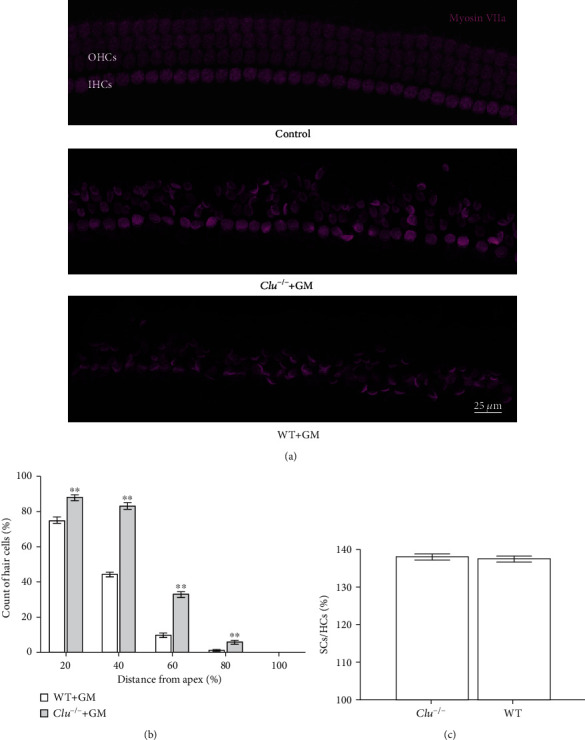
*Clu* deficiency attenuates gentamycin-induced cochlear hair cell death *in vitro*. (a) Representative images of myosin-stained hair cells in the CBM after exposure to the gentamycin (GM). *n* = 6 CBMS per condition. (b) Quantification of myosin staining in IHCs and OHCs. Data are presented as the mean ± SD. ^∗^*p* < 0.05, ^∗∗^*p* < 0.01. *N* = 6. (c) The percentages of supporting cells and hair cells showed no significant differences between WT and *Clu*^−/−^ mice. *N* = 6.

After the hearing test, we used immunohistochemistry to assess hair cell damage. The observed hair cell damage was consistent with the functional deficits indicated by the ABR measurements. The area of the basal turn showed more hair cell loss than the areas of the middle and apical turns, and OHC loss was much greater than IHC loss. In WT and *Clu^−/−^* mice, OHCs completely disappeared from the basal regions of the cochlea; however, the numbers of remaining OHCs in the middle and basal turns were significantly higher in *Clu^−/−^* mice than in WT mice (Figure 6, ^∗^*p* < 0.05), suggesting that *Clu* deficiency protested hair cell death. To confirm protection, we performed experiments in organotypic culture of the organ of Corti in which concentration of gentamicine can be controlled. As shown in Figure 7, treatment with 150 *μ*M gentamicin after 36 hours significantly reduced the number of hair cells in cultured cochlear explants in culture from the WT mouse. The number of remaining hair cells in the cochlea derived from *Clu^−/−^* mice was significantly higher than that in cochlea derived from WT mice. This result was consistent with our in vivo findings, further validating the protective role played by *Clu* deficiency (Figure 7).

## 4. Discussion

HCs in the inner ear cochlea function in transducing sound waves into electric signals [17–21], while supporting cells function in supporting the HCs and providing the potential pool for HC regeneration [22–26]. Damages from a variety of sources can impair HC function, including genetic factors, aging, ototoxic drugs, chronic cochlear infections, and noise exposure [19, 27–31]. In this study, we reported the protective effect of *Clusterin* deficiency against age-related hearing loss and drug-induced ototoxicity, which are both due to irreversible loss of sensory HCs [32–36] and degeneration of the spiral ganglion neurons (SGNs) [37–42]. CLU is an extracellular chaperone protein that has been implicated in diverse physiological and pathophysiological cellular processes. *Clu* expression has been shown to be upregulated in response to cellular stress and under certain environmental conditions, such as during neurodegenerative diseases and cancer. In the current study, we investigated the role of CLU in the inner ear. We found that *Clu* deletion protected cochlear hair cells against aging and ototoxicity, which is the evidence that gene deletion can promote hair cell survival against aging and ototoxicity.

The RNA-seq data showed that *Clu* was expressed in both hair cells and supporting cells [13, 43]. Our RNA-scope results showed that stronger expression was observed in the pillar and Deiters' cells, which is consistent with the results reported by Lee et al. [44]. These differences between the RNA-seq and *in situ* hybridization may be due to difference of sensitivity between RNA-seq and *in situ* hybridization.

We examined hearing thresholds and hair and supporting cell morphology in *Clu^−/−^* mice after birth. Compared with WT mice, the ABR and DPOAE thresholds of the *Clu*^−/−^ mice were not significantly different from those of the WT mice within the first month after birth, which indicated that the deletion of *Clu* did not affect the development of hair cells and supporting cells. This also suggests that the biological processes involved with *Clu* are not related to differentiation and development of hair cells and supporting cells.

ARHL, or presbycusis, is a progressive decline in hearing function and is the most prevalent type of SNHL in the elderly. ARHL is characterized by higher hearing thresholds, beginning at high frequencies and spreading toward low frequencies, accompanied by the loss of HCs and SGNs from the basal to apical turn [45–48]. Surprisingly, we found that deletion of *Clu* slows onset and progression of age-related hair cell and hearing loss.

Clusterin is a glycoprotein that acts as a molecular chaperone to help cells cope with the presence of denatured, misfolded, or aggregated proteins. It often plays a protective role in the pathological processes of various diseases [10, 49]. Our results appeared to be contradictory to the traditional view of CLU function. However, studies of *Clu* mutations in Alzheimer's disease have suggested that CLU is associated with a high risk of late-onset Alzheimer's disease [50, 51]. No studies have been done to examine the relationship between CLU and hearing.

Many stress stimulators like ototoxic drugs had been testified to vast ROS production in HCs [52–58]. After observing the protective effects of *Clu* deficiency against ARHL and considering the importance of hair cells in drug-induced deafness, we evaluated whether *Clu* deficiency could confer protection against aminoglycoside ototoxicity by performing both *in vivo* and *in vitro* experiments. Our results showed that *Clu* deficiency protected hair cell death. The sensory hair cells of the inner ear are the primary target of aminoglycoside ototoxicity, and no therapies are currently available that can prevent ototoxicity. The mechanism through which *Clu* deficiency protects hair cells from ototoxic drugs remains unclear.

We speculate that the protective effects of *Clu* deficiency in hair cells may be the result of direct effects on hair cells, indirect effects mediated by supporting cells, or a combination of effects associated with both hair cells and supporting cells. Supporting cells may exert various effects on hair cells by providing an environment where hair cells can live [59, 60]. Some recent studies have shown that supporting cells may promote hair cell repair when *Atoh1* is overexpressed in Deiters' cells [61, 62]. Deiters' cells may also release organelles to promote survival of hair cells in response to stress [63].

In summary, our results revealed the protective effects of *Clu* deficiency in cochlear hair cells. Thus, *Clu* may be a good target gene for therapeutic interventions to slow ARHL and prevent ototoxic drug-induced hearing loss. Future work is necessary to determine the molecular mechanism through which *Clu* deletion protects against age-related hair cell apoptosis and aminoglycoside-induced hair cell loss.

## Data Availability

The data used to support the findings of this study are included within the article, and the data are available.
